# Influencers of the Decision to Undergo Contralateral Prophylactic Mastectomy among Women with Unilateral Breast Cancer

**DOI:** 10.3390/cancers13092050

**Published:** 2021-04-23

**Authors:** Akshara Singareeka Raghavendra, Hala F. Alameddine, Clark R. Andersen, Jesse C. Selber, Abenaa M. Brewster, Carlos H. Barcenas, Abigail S. Caudle, Banu K. Arun, Debu Tripathy, Nuhad K. Ibrahim

**Affiliations:** 1 Department of Breast Medical Oncology, Division of Cancer Medicine, The University of Texas MD Anderson Cancer Center, Houston, TX 77030, USA; asraghavendra@mdanderson.org (A.S.R.); abrewster@mdanderson.org (A.M.B.); CHBarcenas@mdanderson.org (C.H.B.); barun@mdanderson.org (B.K.A.); dtripathy@mdanderson.org (D.T.); 2 Kindred Hospital Department of Nursing, Sugar Land, TX 77479, USA; Hala.Alameddine@kindred.com; 3 Department of Biostatistics, The University of Texas MD Anderson Cancer Center, Houston, TX 77030, USA; crandersen@mdanderson.org; 4 Department of Plastic Surgery, The University of Texas MD Anderson Cancer Center, Houston, TX 77030, USA; JCSelber@mdanderson.org; 5 Department of Surgery, The University of Texas MD Anderson Cancer Center, Houston, TX 77030, USA; ascaudle@mdanderson.org

**Keywords:** contralateral prophylactic mastectomy, breast cancer, contralateral breast cancer, unilateral breast cancer

## Abstract

**Simple Summary:**

In this survey study, we examined survey responses from 397 women with stage 0 to III unilateral breast cancer and found that partners, physicians, and the media were significant relative to the patient’s own influence in their decision to undergo a CPM. The findings of this study may inform policy by highlighting the need for educational aids, programs, or tools that help women with unilateral breast cancer make informed, evidence-based decisions regarding CPM efficacy.

**Abstract:**

(1) Background: The relatively high rate of contralateral prophylactic mastectomy (CPM) among women with early stage unilateral breast cancer (BC) has raised concerns. We sought to assess the influence of partners, physicians, and the media on the decision of women with unilateral BC to undergo CPM and identify clinicopathological variables associated with the decision to undergo CPM. (2) Patients and Methods: Women with stage 0 to III unilateral BC who underwent CPM between January 2010 and December 2017. Patients were surveyed regarding factors influencing their self-determined decision to undergo CPM. Partner, physician, and media influence factors were modeled by logistic regressions with adjustments for a family history of breast cancer and pathological stage. (3) Results: 397 (29.6%) patients completed the survey and were included in the study. Partners, physicians, and the media significantly influenced patients’ decision to undergo CPM. The logistic regression models showed that, compared to self-determination alone, overall influence on the CPM decision was significantly higher for physicians (*p* = 0.0006) and significantly lower for partners and the media (*p* < 0.0001 for both). Fifty-nine percent of patients’ decisions were influenced by physicians, 28% were influenced by partners, and only 17% were influenced by the media. The model also showed that patients with a family history of BC had significantly higher odds of being influenced by a partner than did those without a family history of BC (*p* = 0.015). (4) Conclusions: Compared to self-determination, physicians had a greater influence and partners and the media had a lower influence on the decision of women with unilateral BC to undergo CPM. Strong family history was significantly associated with a patient’s decision to undergo CPM.

## 1. Introduction

Several clinical and pathological factors may be related to an increased risk for developing contralateral breast cancer in women with unilateral breast cancer. Some of the known risk factors include young age at primary breast cancer diagnosis, a family history of breast cancer, having an estrogen receptor-positive primary tumor, and having a *BRCA* mutation [1]. However, for most women with early stage, sporadic, unilateral breast cancer, the cumulative lifetime risk of CBC at 5, 10, 15, and 20 years was 3%, 6.1%, 9.1%, and 12%, respectively [2].

Women with high risk of developing breast cancer may undergo a prophylactic mastectomy. In addition, women who are diagnosed with unilateral breast cancer and have a high risk of developing contralateral breast cancer may consider risk reduction of contralateral breast cancer. The standard-of-care recommendation is that contralateral prophylactic mastectomy (CPM) be performed on women with unilateral breast cancer and *BRCA* mutations [3,4,5], with no demonstrable clinical benefit in strong family history or young age at presentation [6].

However, some women with unilateral breast cancer choose to undergo CPM when it is not clinically indicated for many reasons. Patients hope to avoid “cumbersome” repeated breast imaging and associated anxiety and achieve body image symmetry short of reconstructive options, fear of the risk of contralateral cancer and potential need for further systemic therapy, influences of acquaintances or celebrities undergoing CPM covered in the media, and lack of knowledge regarding outcome data for their situation [3,7].

The increasing rate of CPM among women with unilateral breast cancer, from 3.9% to 12.7% from 2002 to 2012, at an early stage and with no clinical indications for CPM, has raised concern among treating physicians [8]. Questionnaire-based data from the ‘‘Helping Ourselves, Helping Others: Young Women’s Breast Cancer Study’’ (YWS) reported that 60% of CPM recipients had negative testing for *BRCA* mutations, and 70% did not have a positive family history of breast cancer within a first-degree relative [9,10]. A study conducted in our institution reaffirmed that the rate of CPM was independent of *BRCA* carrier status and that non-*BRCA* genes and variants do steer women to undergo CPM [11]. CPM rate for DCIS, considered as stage 0, increased from 5.4% to 37.5% from 1998 to 2011 [12]. This is particularly noteworthy as there is no evidence of a survival benefit from the CPM in this setting [13] and because the risk of developing contralateral breast cancer is 0.5–0.75% per year among women with unilateral, early stage, sporadic breast cancer [14]. Although CPM does not improve survival, many women with unilateral breast cancer undergo the procedure due to fear of recurrence or the expectation that this may extend their lives [10].

Shared decision making, entailing the clinicians and patients working together on care plans based on clinical evidence, can balance risks and expected outcomes with patient preferences and values [15]. The purpose of this survey study was to examine the influence of partners, physicians, and audio-visual and printed media, in addition to all forms of social media, on the decision making of women with unilateral, early stage breast cancer to choose CPM and to analyze the clinicopathological variables associated with a patient’s decision to undergo CPM. We specifically chose to examine the influence of partners, physicians, and the media because few studies have examined their impact on women’s decision to undergo CPM.

## 2. Materials and Methods

Using the prospectively maintained Breast Cancer Database Management System housed and curated in the Department of Breast Medical Oncology at the University of Texas MD Anderson Cancer Center, we identified women diagnosed with early stage breast cancer (stage 0-III) who underwent CPM between January 2010 and December 2017 with no clinical or radiographic evidence of contralateral breast cancer. Excluded from the study were women who had bilateral breast cancer before undergoing CPM, had received any treatment for breast cancer before their initial visit to MD Anderson Cancer Center, were *BRCA1* or *BRCA2* mutation carriers as well as other relevant mutations including *CHEK2, TP53, ATM, PALB2, PTEN,* and *CDH1*, which are associated with familial breast cancers, or had presented for a second opinion and not to pursue management at our center [16].

Patient characteristics retrieved from the medical records included age at the time of breast cancer diagnosis, body mass index, and significant family histories of breast cancer (with any 1st or 2nd degree relative with breast cancer). We also recorded tumor characteristics, including histology (infiltrating ductal carcinoma versus infiltrating lobular carcinoma versus mixed), estrogen/progesterone/HER2 receptor status, and axillary nodal status.

Patients received an emailed link to a quantitative, cross-sectional survey (Appendix A) consisting of 16 questions adopted with modification from the Prophylactic Mastectomy Outcomes Study Survey [17]. This questionnaire was adapted to analyze our hypothesis, which may need to be verified in future studies. Through the program used to send the emailed surveys, we were able to determine if respondents had responded to the survey, opted out, or if the emailed survey was undeliverable. Patients received emails reminding them to complete the survey after 2 and 4 days. No clinical data were collected for patients who did not consent to the survey.

The survey design provided a numeric rating of the influence of partners, physicians, and the media on patients’ decisions to undergo CPM. The survey allowed the patients to select “no response” or “prefer not to respond” in response to every survey question, which were considered completed responses. This study was needed because breast cancer patients may be influenced by others when deciding whether to undergo CPM. Survey data were collected and managed using REDCap electronic data capture tools hosted at MD Anderson [18]. MD Anderson’s Institutional Review Board approved the informed consent document and survey; the informed consent document was included as part of the survey package.

Descriptive statistics were used to summarize patient characteristics.

To assess the influence of partners, physicians, and the media on patients’ decisions to undergo CPM, three binary outcome variables were defined. Doctor-influenced versus self-choice (implying that the patient had checked at least one of the “doctor”-related influence statements in the survey (doctor-influenced) versus having checked the “I made the final decision to have surgery” but none of the doctor-influenced statements (self-choice)), Partner-influenced versus self-choice (implying that the patient had checked at least one of the “partner”-related influence statements in the survey (partner-influenced) versus having checked the “I made the final decision to have surgery” but none of the partner-influenced statements (self-choice), for patients who indicated the presence of partners under the Marital Status section of the survey), Media-influenced versus self-choice (implying that the patient had checked at least one of the “media”-related influence statements on the survey other than “not at all” (media-influenced) versus having checked the “I made the final decision to have surgery” but none of the media-influenced statements (self-choice)).

The incidence of each of the binary outcomes was modeled by a logistic regression (a binomial distribution with a logit link). For each outcome, the inclusion of potential covariates was assessed by adding them to the model and comparing them to the model without the covariate using the Akaike information criterion. Potential covariates considered included the presence of a family history of breast cancer, age category, race, marital status, presence of a partner, education category, presence of estrogen or progesterone receptor, presence of lymph vascular invasion (LVI), whether the CPM was performed on a different versus the same day as the definitive surgery, tumor grade, and pathology stage. The covariates for marital status and the presence of a partner were excluded from the partner-influenced model due to confounding with partner influence. Balancing the model selection among the outcomes, together with that of a model that combined the outcomes as an any-influence model (results not reported), a consensus logistic regression model was selected that included a family history of breast cancer and pathological stage as covariates. For consistency, the final analysis summaries and figures for all outcomes were based on these covariate models. Differences among levels of pathology stage were assessed by Tukey-adjusted contrasts.

Statistical analyses were performed using R statistical software [19]. In all statistical tests, a two-sided alpha = 0.05 was considered statistically significant. Predictions and differences among factor levels in the logistic regression models were estimated using the emmeans package version 1.4.2 [20]; adjusted means were weighted proportionally to covariate marginal frequencies. Cat’s eye plots [21] were produced using the catseyes package version 0.2.2 [22].

## 3. Results

**Figure 1:**
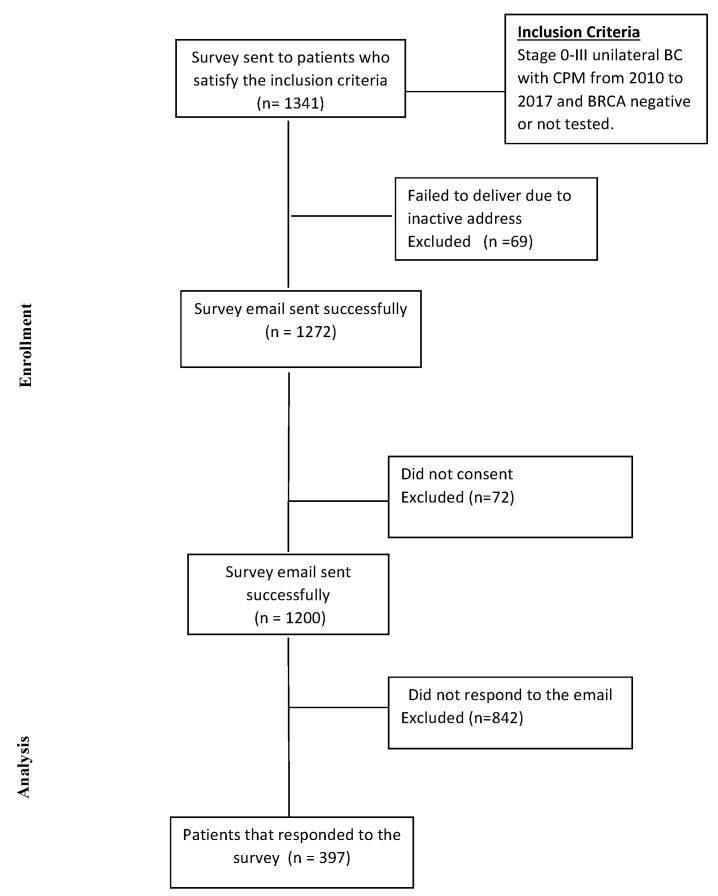
Consort diagram. BC = breast cancer; CPM = contralateral prophylactic mastectomy.

In MD Anderson’s electronic database, we identified 1341 patients with stage 0-III breast cancer who were eligible for the survey (Figure 1).

Of these, 397 (29.6%) responded to the survey (summarized in Table 1 and Table 2).

Two hundred and eight (56.1%) were concerned about developing breast cancer, 163 (43.9%) were not concerned, and 28 (7%) did not respond about developing breast cancer before undergoing CPM.

Of the 343 patients with complete responses regarding physician influence (see Table 2), 203 (59%) reported a physician’s influence on their decision to undergo CPM. The logistic regression model (Table 3) showed that, compared to self-determination alone, the overall influence of physicians on patients’ CPM decisions was significantly higher (*p* = 0.0006).

**Figure 2:**
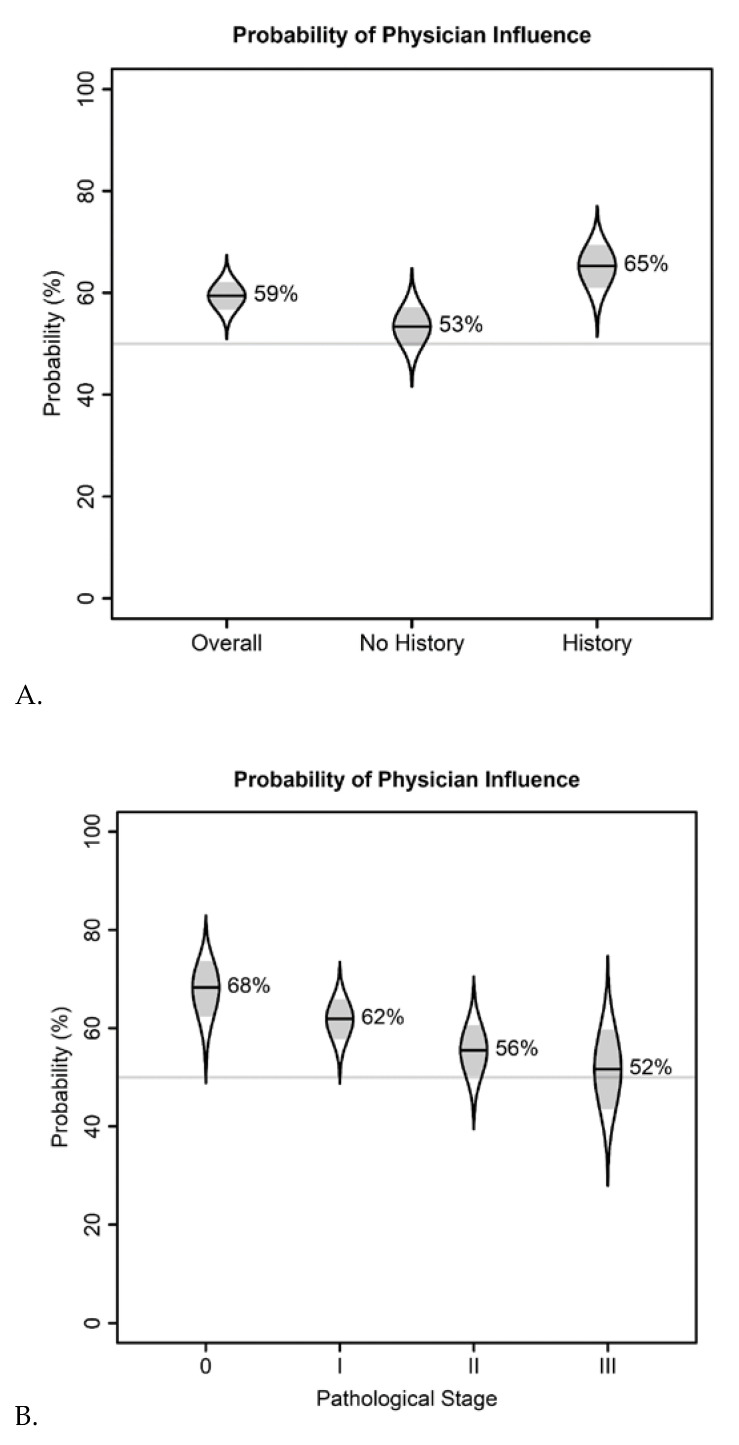

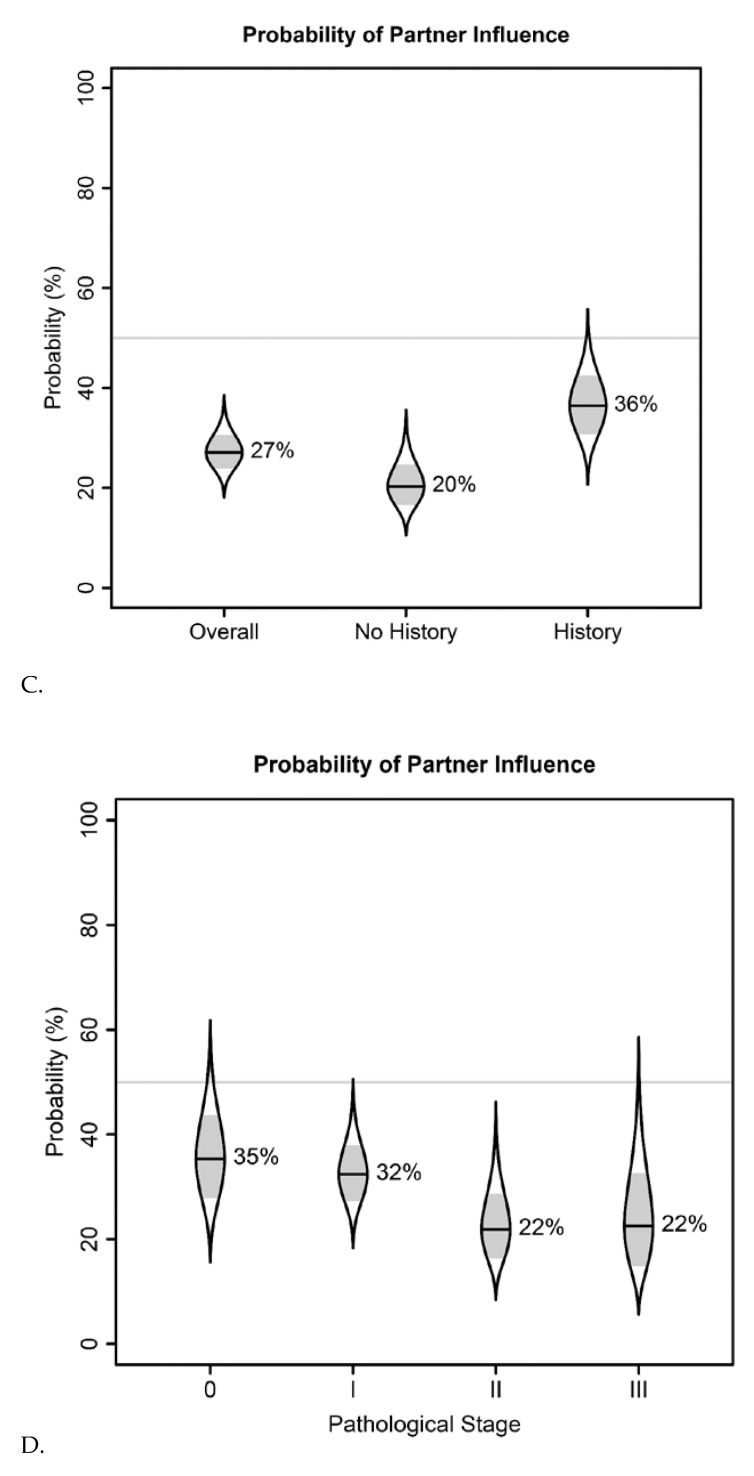

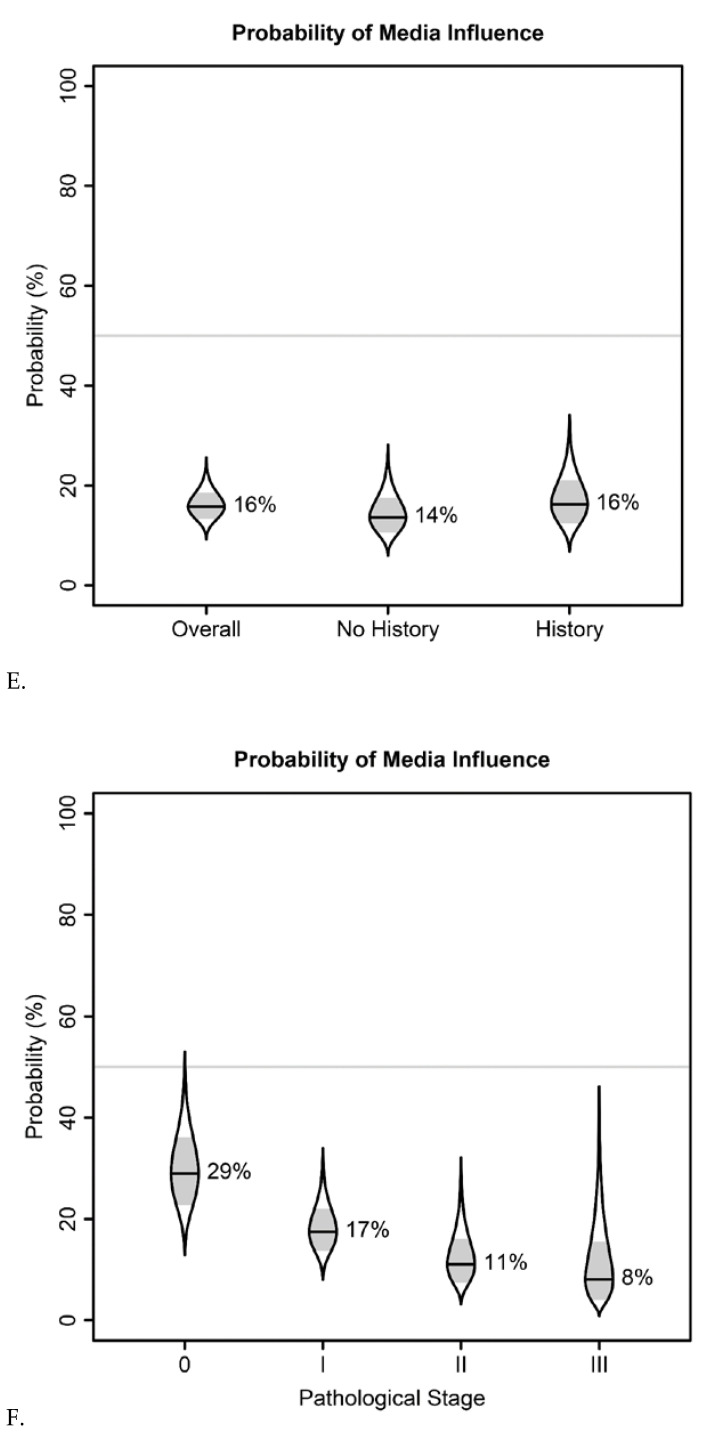
Cat’s eye plots of logistic regression results show the probabilities of breast cancer patients’ CPM decisions being influenced by (**A**) physicians, overall and according to patients’ family histories of breast cancer; (**B**) physicians, according to patients’ pathological breast cancer stages; (**C**) partners, overall and according to patients’ family histories of breast cancer; (**D**) partners, according to patients’ pathological breast cancer stages; (**E**) the media, overall, and according to patients’ family histories of breast cancer; and (**F**) the media according to patients’ pathological breast cancer stages. The distributions of the model-adjusted means have been transformed from the logit scale to the probability scale; distributions near 0% or 100% have been distorted accordingly. The horizontal lines in the cat’s eye plots indicate 50% probabilities, and standard errors are shaded.

The model also showed that patients with a family history of breast cancer had significantly higher odds of being influenced by a physician than did those without a family history of breast cancer (*p* = 0.029). There was no evidence of a significant association between pathological stage and physician influence. These results are summarized in Table 3 and Figure 2.

Of the 189 patients with complete responses regarding partner influence (see Table 2), 53 (28%) reported a partner’s influence on their decision to undergo CPM. The logistic regression model showed that, compared to self-determination alone, the overall influence of partners on patients’ CPM decisions was significantly lower (*p* < 0.0001). The model also showed that patients with a family history of breast cancer had significantly higher odds of being influenced by a partner than did those without a family history of breast cancer (*p* = 0.015). There was no evidence of a significant association between pathological stage and partner influence. These results are summarized in Table 3 and Figure 2.

Of the 213 patients with complete responses regarding the influence of the media (see Table 2), 36 (17%) reported that the media influenced their decision to undergo CPM. The logistic regression model showed that, compared to self-determination alone, the overall influence of the media on patients’ CPM decisions was significantly lower (*p* < 0.0001). The model also showed that the odds of being influenced by the media were higher in patients with a family history of breast cancer than in those without a family history of breast cancer, but the difference was not statistically significant (*p* = 0.059). There was no evidence of a significant association between pathological stage and media influence. These results are summarized in Table 3 and Figure 2A–F.

Although the associations between physician, partner, and media influences and pathological stage (stages 0-III) lacked statistical significance, as the pathological stage increased, there was a trend of a declining probability of physician, partner, and media influence (Figure 1).

Most women (83.3%) were satisfied with their CPMs. A smaller number were neutral (7.2%) or dissatisfied (9.4%).

Our overall study findings are summarized in Table 4.

## 4. Discussion

The results of this study indicated that women with early stage, unilateral breast cancer who underwent CPM, although the procedure was not clinically indicated, did not make the decision to undergo CPM alone. A key finding of this study is that partners, physicians, and the media all significantly influenced the decision of women with unilateral breast cancer to undergo CPM.

In a single institution study of 2504 patients with early breast cancer (stage 0-III) who had breast-conserving surgery or mastectomy for their primary tumor, 11.3% had, in addition, CPM [18]. Patients who had mastectomy were included in the study, but we did not address whether or not the patients were recommended BCS. Previous studies did find that prior BCS followed by bilateral mastectomies was common in the CPM group (28%) [23,24]. Clinicopathologic characteristics associated with undergoing CPM included a family history of breast cancer, age at diagnosis of breast cancer, white race, tested for *BRCA1* or *BRCA2* mutation, clinical tumor stage, and lobular histology as well as patients who underwent reconstructive surgery [25]. In addition, of the 33 patients who were tested for BRCA mutations, all eight patients who carried a mutation had CPM; on the other hand, 10/25 (40%) patients who were negative for *BRCA* mutations had CPM [18]. The influence of the other clinical factors on this small group of patients is not clear. Therefore, the decision for CPM was seemingly multifactorial, as the majority of the patients who had CPM did not have genetic testing.

Among the surveyed patients with *BRCA* non-mutations, only a positive family history seemed to have a significant bearing on the patient’s decision to proceed with CPM. In contrast, other studies showed the rates of CPM among patients with no family history of breast cancer or *BRCA* mutations was 60–70% [5,6]. Even in patients at stage 0, the incidence of CPM was 5.4–35% [26]. The seeming lack of association between the decision to undergo CPM with tumor or treatment characteristics that may suggest an increased risk of contralateral breast cancer supports the idea that patients may choose to undergo CPM for other perceived reasons of potential relevance. We found that women who underwent CPM did not decide on their own whether to undergo the procedure but were influenced by their partners, physicians, and the media. These results suggest that both clinical and non-clinical factors motivate patients to consider CPM.

While our patients were selected sequentially from the database, the majority were white educated women. A study by Tuttle et al. [27] indicated that being younger than 45 years and white were associated with the decision to undergo CPM. In addition, a population-based study found that having estrogen receptor-positive breast cancer and having stage I or II disease were associated with the decision to undergo CPM [28]. However, the risk of recurrence from the primary cancer is greater than the risk of developing contralateral breast cancer, with no apparent survival benefit associated with CPM [27].

The finding that patients with a family history of breast cancer were more susceptible to influence by a partner than those without a family history of breast cancer to make the decision to undergo CPM is consistent with other studies [29]. These women may not have been able to make an objective, informed decision regarding CPM because of the benefits versus the risks of the procedure, or due to their own interpretation of factual or unrelated information they may have acquired otherwise. A recent study highlights an important finding that young age or strong family history without genetic mutations had less demonstrable benefit of CPM compared to those with demonstrable genetic mutations [6].

Indeed, the Society of Surgical Oncology suggests that CPM should be considered in patients with (1) a *BRCA1* or *BRCA2* mutation or strongly predisposing breast cancer susceptibility genes or (2) a strong family history of at least two first-degree relatives with breast or ovarian cancer and with no demonstrable mutations [7].

When faced with life-threatening diseases like breast cancer, patients might make uninformed decisions regarding their treatment [30] They might also overestimate the benefits of CPM, thinking that the procedure will reduce their contralateral breast cancer risk and confer a survival benefit [31,32,33] Others may underestimate the severity of some of the side effects associated with CPM. On the other hand, CPM may be associated with patient’s satisfaction with their breasts but not with improvements in other health-related quality of life issues [25]. Reasons given for satisfaction include peace of mind [34,35,36], satisfaction with cosmetic results [34] and body image [36,37], an absence of problems with the procedure [34], risk reduction [34], and a sense of “prevailing over cancer” [36].

It is important, therefore, for women with unilateral breast cancer to fully understand and quantify the benefits and side effects associated with CPM, and help them adjust their expectations of the outcomes, if necessary, all in the context of their personal need and sociocultural standing [33]. To accomplish this goal, patients should be provided with decision aids, where available, such as informative brochures, videos, and computer programs where physicians share information and patients can express their preferences regarding their treatment options during the decision-making process [38].

The binary influence variables that formed the basis of these analyses were not independent. Most patients reporting influence from partners or the media also report influence from physicians. Physicians often provide clear and helpful information that define the roles, responsibilities, and expectations in this asymmetrical relationship [39]. Two-thirds of patients reported that their decision was subject to some form of influence. Taken together, our results suggest that a patient with a family history of breast cancer is more likely to consider external perspectives when deciding whether to undergo CPM. This research includes a large well-annotated database with variables such as *BRCA* testing, a structured questionnaire, and a statistical approach by influencer effect. Some of the potential limitations include adaptation of a modified validated questionnaire without further affirmation, which might impact the validity of the results, population bias, recall bias as the study identified patients over an 8-year period, and a lack of a control group (that did not choose CPM).

A potential contribution of our study is that it advocates positive interactions between women with unilateral breast cancer and their partners and physicians, as well as the media to pair with awareness of real indications and benefits of CPM as it is not a good decision for every patient, and rather to promote a methodological, personalized medical decision approach as they decide whether to undergo CPM. Our results also highlight the need for decision-making aides, programs, tools, and other innovations from the burgeoning field of decision science [40] to help women with breast cancer increase their knowledge of their treatment options and make informed decisions that align with their goals and values.

## 5. Conclusions

In conclusion, compared to self-determination, physicians had a greater influence and partners and media had a lower influence on the decisions of women with unilateral BC to undergo CPM.

Shared decision making involving patients and physicians conducted soon after patients’ breast cancer diagnoses would help women decide which medical treatment option is best for them based on current evidence. A clinical educational instrument would also help women with unilateral breast cancer make informed decisions regarding CPM. It is important for women with unilateral breast cancer to fully understand the benefits and adverse effects of CPM and make an informed decision regarding this irreversible surgical procedure.

## Figures and Tables

**Table 1:** Demographic characteristics of study participants.

Demographic	N (%)
Age at Diagnosis of Breast Cancer	
20 to 30	16 (4)
31 to 40	104 (26)
41 to 50	169 (43)
51 to 60	108 (27)
Race	
Asian or Pacific Islander	14 (4)
Black or African American	15 (4)
Hispanic/Latino	35 (9)
Native American or Alaskan	1 (0)
White or Caucasian	328 (83)
Other	4 (1)
Education Level	
Less than or some high school	0 (0)
High school or general educational development	29 (7)
Trade or technical school	13 (3)
Junior college or some college	64 (16)
College graduate	132 (33)
Post-graduate work or degree	120 (30)
No response	39 (10)
Marital status	
Married	305 (77)
Living together but not married	12 (3)
Separated or divorced	28 (7)
Widowed	10 (3)
Never married	18 (5)
No response	24 (6)
Stage	
I	152 (38)
II	181 (46)
III	64 (16)
Grade	
I	28 (7)
II	160 (40)
III	209 (53)
Family History	
Reported	170 (43)
Not reported	227 (57)
Total participants responded to survey	397(100)

**Table 2:** The effect of self-determination, partners, physicians, and the media on women’s decisions to undergo contralateral prophylactic mastectomy. The N (%) column gives counts with percentages by decision, with the total count at the bottom; note that patients could select multiple decisions, so the sum of counts exceeds the total. The Physician, Partner, and Media columns indicate responses which contributed to determination of the respective influences with an “X”, which require non-missing responses among those contributing, as described in the Patients and Methods section. The “Count with influence other than self” row provides the count (percentage) of respondents indicating each respective influence, with percentage out of the respective total. The Total row for the Physician, Partner, and Media columns gives the total number of respondents who either indicated that they had made the final decision alone or reported some influence.

Decision	N (%)	Physician	Partner	Media
I made the final decision to have surgery.	201 (54)	X	X	X
I made the final decision to have surgery after seriously considering my doctor’s opinion.	165 (44)	X		
My doctor and I shared responsibility for the final decision to have surgery.	60 (16)	X		
My doctor made the final decision about my surgery, but seriously considered my opinion.	2 (1)	X		
My doctor made the final decision about my surgery.	4 (1)	X		
I made the final decision to have surgery after seriously considering my partner’s opinion.	59 (16)		X	
My partner made the final decision about my surgery.	1 (0)		X	
Media Influence: Please choose one number to indicate whether or not the media had influenced your decision making to undergo prophylactic mastectomy (count indicates any influence other than “Not at all”)	37 (10)			X
Count with influence other than self (combines multiple questions)		203 (59)	53 (28)	36 (17)
Total	373	343	189	213

**Table 3:** Logistic regression summary of the influence of physician, partner, and the media on CPM. This table summarizes the results of 3 separate logistic regression models, which separately modeled the incidence of physician, partner, or media influence. Each model also controlled for (included as covariates) family history of breast cancer and pathology stage to improve the model. The table shows the model-adjusted probability of reporting each type of influence. The relationship between presence of family history and each type of influence is also reported as odds ratios; the presence of family history corresponded to higher odds of reporting each type of influence, though it lacked significance for media influence. Results for pathology stage lacked significant evidence of association and are not shown.

	Physician Influence	Partner Influence	Media Influence
Overall probability of influence on cpm decision (95% CI)	59% (54–65%)	27% (21–34%)	16% (11–22%)
	*p* = 0.0006	*p* < *0*.0001	*p* < 0.0001
Odds of influence on CPM decision given family history of breast cancer (95% CI)	1.64 (1.05–2.57)	2.25 (1.17–4.34)	1.23 (0.59–2.58)
	*p* = 0.029	*p* = 0.015	*p* = 0.059

Tukey-adjusted *p* value.

**Table 4:** Summary of Overall Study findings.

Influencers	Influenced Decision to Undergo CPM	*p*-Value
Partners	28%	<0.0001
Physicians	59%	0.006
Media	17%	<0.0001

## Data Availability

The data that support the findings of this study are available from the corresponding author, upon request.

## Appendix A

Contralateral Prophylactic Mastectomy Survey

Living with Breast Cancer Risk: Survey of Experiences and Decision-Making Process

Please check the one best answer to each of the following questions, unless instructed otherwise.

**Your Breast Cancer Experience and Thoughts**

1. Before your contralateral prophylactic mastectomy, how would you have described your concern about developing breast cancer?
  4□ Very concerned
  3□ Concerned
  2□ Not very concerned
  1□ Not concerned at all
2. At the time of your prophylactic mastectomy, what was your marital status?
  1□ Married
  2□ Living together but unmarried
  3□ Separated or divorced
  4□ Widowed
  5□ Single, never married
3. What were your reasons for having a contralateral prophylactic mastectomy? Please check all that apply.
  1□ Uncomfortably large breasts
  2□ Concerns about appearance
  3□ Family history of breast cancer
  4□ Prevent breast cancer
  5□ Other, please specify: __________________________________________
4. Which statement (s) best describes the decision about your contalateral prophylactic mastectomy? Choose all that apply.
  1□ I made the final decision to have surgery.
  2□ I made the final decision to have surgery after seriously considering my doctor’s opinion.
  3□ My doctor and I shared responsibility for the final decision to have surgery.
  4□ My doctor made the final decision about my surgery, but seriously considered my opinion.
  5□ My doctor made the final decision about my surgery.
  6□ I made the final decision to have surgery after seriously considering my partner’s opinion.
  7□ My partner made the final decision about my surgery.
5. Media Influence: Please choose one number to indicate whether or not the media had influenced your decision making to undergo prophylactic mastectomy.
  **Not At All**	**A Little Bit**	**Some-What**	**Quite A Bit**	**Very Much**
  1	2	3	4	5
6. Thinking back to six months after your prophylactic mastectomy, how satisfied were you with your decision to have the surgery?
  1□ Very dissatisfied
  2□ Dissatisfied
  3□ Neither Satisfied or Dissatisfied
  4□ Satisfied
  5□ Very satisfied
7. Did you have breast reconstruction after your prophylactic mastectomy? Breast reconstruction is a surgical procedure in which the breasts are recreated using implants or tissue from the body.
  0□ No.
  1□ Yes, done in a separate surgery after the prophylactic mastectomy
  2□ Yes, done along with prophylactic mastectomy
8. I “yes” Have you had surgery to revise or repair your reconstruction?
  0□ No
  1□ Yes, one or two times
  2□ Yes, multiple times

**Your Life Right Now**

9. Below is a list of statements that describe aspects of women’s lives, including thoughts about your body and sexuality.
  **Please Choose One Number to Indicate How True Each Statement Has Been for You during the Past 30 Days.**	**FREQUENCY**				
  	**Not At All**	**A Little Bit**	**Some-What**	**Quite A Bit**	**Very Much**
  a. I am able to enjoy life.	1	2	3	4	5
  b. I am content with the quality of my life right now.	1	2	3	4	5
  c. I feel self-conscious about my appearance.	1	2	3	4	5
  d. I am happy with my current weight.	1	2	3	4	5
  e. I am satisfied with my appearance when dressed.	1	2	3	4	5
  f. I find it difficult to look at myself naked.	1	2	3	4	5
  g. I am embarrassed for others to see my body.	1	2	3	4	5
  h. I am able to feel like a woman.	1	2	3	4	5
  i. I feel sexually attractive.	1	2	3	4	5
  j. I am satisfied with my sex life.	1	2	3	4	5

**A Few Details About You**

10. What was your age at the time of prophylactic mastectomy?
  1□ 20 to 30 years old
  2□ 31 to 40 years old
  3□ 41 to 50 years old
  4□ 51 to 60 years old
11. To what race/ethnic group do you belong? Please check all that apply.
  1□ Asian or Pacific Islander, please specify: ________________________
  2□ Black or African American
  3□ Hispanic/Latino, please specify:________________________
  4□ Native American or Alaskan Native
  5□ White or Caucasian
  9□ Other, please specify: ________________________
12. What is the highest level of education you have completed?
  1□ Less than or some high school
  2□ High school or GED
  3□ Trade or technical school
  4□ Junior college, or some college
  5□ College graduate
  6□ Postgraduate work or degree
13. On what date did you complete this questionnaire?____/____/____ month/day/year)
14. How long ago was your prophylactic mastectomy? (Please insert the number of years)___________Years ago.

**Final Questions**

15. Overall, how satisfied are you now with your decision to have contralateral prophylactic mastectomy?
  1□ Very dissatisfied
  2□ Dissatisfied
  3□ Neither Satisfied or Dissatisfied
  4□ Satisfied
  5□ Very satisfied
16. What one thing do you wish you had known before your prophylactic mastectomy?________________________________________________________________________________________________________________________________________________________________________________________________________________________
